# Abnormal wiring of the structural connectome in adults with ADHD

**DOI:** 10.1162/netn_a_00326

**Published:** 2023-12-22

**Authors:** Tuija Tolonen, Timo Roine, Kimmo Alho, Sami Leppämäki, Pekka Tani, Anniina Koski, Matti Laine, Juha Salmi

**Affiliations:** Department of Psychology and Logopedics, University of Helsinki, Helsinki, Finland; Department of Neuroscience and Biomedical Engineering, Aalto University, Espoo, Finland; Turku Brain and Mind Center, University of Turku, Turku, Finland; AMI Centre, Aalto Neuroimaging, Aalto University, Espoo, Finland; Terveystalo Healthcare, Helsinki, Finland; Department of Psychiatry, Helsinki University Hospital, Helsinki, Finland; Department of Psychology, Åbo Akademi University, Turku, Finland

**Keywords:** Adult ADHD, Diffusion, Graph theory, Network-based statistic, Connectivity, Symptoms

## Abstract

Current knowledge of white matter changes in large-scale brain networks in adult attention-deficit/hyperactivity disorder (ADHD) is scarce. We collected diffusion-weighted magnetic resonance imaging data in 40 adults with ADHD and 36 neurotypical controls and used constrained spherical deconvolution–based tractography to reconstruct whole-brain structural connectivity networks. We used network-based statistic (NBS) and graph theoretical analysis to investigate differences in these networks between the ADHD and control groups, as well as associations between structural connectivity and ADHD symptoms assessed with the Adult ADHD Self-Report Scale or performance in the Conners Continuous Performance Test 2 (CPT-2). NBS revealed decreased connectivity in the ADHD group compared to the neurotypical controls in widespread unilateral networks, which included subcortical and corticocortical structures and encompassed dorsal and ventral attention networks and visual and somatomotor systems. Furthermore, hypoconnectivity in a predominantly left-frontal network was associated with higher amount of commission errors in CPT-2. Graph theoretical analysis did not reveal topological differences between the groups or associations between topological properties and ADHD symptoms or task performance. Our results suggest that abnormal structural wiring of the brain in adult ADHD is manifested as widespread intrahemispheric hypoconnectivity in networks previously associated with ADHD in functional neuroimaging studies.

## Author Summary

Although it is well-established that widespread changes in large-scale brain networks underlie ADHD, little is known about the aberrancies in the complex structural brain networks that pertain at adulthood. Knowledge of structural changes in adult ADHD would be important as the symptom presentation of the disorder changes considerably across the life-span. We found that abnormal structural brain wiring in adults with ADHD is manifested as widespread hypoconnectivity in unilateral networks, and attention task performance is associated with a left-frontal network across all participants. White matter abnormalities in overlapping brain networks have been reported also in children with ADHD, but emerging evidence in adults proposes some changes in the neuropathology of ADHD from childhood to adulthood.

## INTRODUCTION

Attention-deficit/hyperactivity disorder (ADHD) is a neurodevelopmental disorder characterized by inattention, impulsivity, and hyperactivity (American Psychiatric Association, 2013). About 65% of the individuals who receive ADHD diagnosis at childhood continue to have difficulties with attention at adulthood (Faraone et al., 2006; Simon et al., 2009). Although it is well-established that widespread changes in large-scale brain networks underlie ADHD (Cao et al., 2014; Castellanos & Aoki, 2016; Konrad & Eickhoff, 2010), little is known about the aberrancies in the complex structural brain networks that pertain at adulthood. Further knowledge of structural changes in adult ADHD would be important as the symptom presentation of the disorder changes considerably across the life-span (Vos et al., 2022).

Connectionist approach focusing on the role of the complex brain wiring in typical and atypical development has been rapidly advancing, in parallel with the development of diffusion-weighted magnetic resonance imaging (DW-MRI; Le Bihan et al., 2001), and resting-state functional MRI (rs-fMRI; Biswal et al., 1995). In individuals with ADHD, abnormal brain structure and function have been reported in various areas, including each cerebral lobe, and several subcortical structures, such as the basal ganglia and cerebellum (Cortese et al., 2012; Frodl & Skokauskas, 2012; Norman et al., 2016). Many of the DW-MRI findings, especially in adults, are based on testing differences in white matter fractional anisotropy in regions of interest or skeletonized tracts (Aoki et al., 2018; Bouziane et al., 2017; Konrad et al., 2010; Onnink et al., 2015). A recent meta-analysis by Aoki and colleagues (2018), including studies on both children and adults, suggested decreased fractional anisotropy in participants with ADHD in the inferior fronto-occipital and occipito-temporal fasciculi and callosal pathways, as compared with neurotypical (NT) controls. Although a majority of the studies included in this meta-analysis were performed in children and adolescents, altered white matter structures in largely overlapping brain areas have been reported in studies focusing on adult population (Bode et al., 2015; Chaim et al., 2014; Cortese et al., 2013; Konrad et al., 2010; Onnink et al., 2015). Data-driven whole-brain analysis techniques focusing on properties of networks connecting multiple brain areas and graph theoretical analysis that aims to characterize the large-scale topological properties of the brain networks have recently become increasingly common also in ADHD research (Connaughton et al., 2022).

In network-based statistics (NBS), whole-brain connectivity networks are divided into lower level subnetworks reflecting group differences without a priori information of their distribution or size (Zalesky et al., 2010). To our knowledge, so far only two studies of adult ADHD have utilized NBS together with DW-MRI. He and colleagues (2022) found increased connectivity in the ADHD group in relation to NT controls between several regions including subcortical structures such as amygdala, thalamus and putamen, and medial, frontal and orbital cortical regions. On the other hand, Hearne and colleagues (2021) found no significant networks differentiating adults with ADHD from NT controls. Increased connectivity in adults with ADHD has also been found in a study utilizing resting-state functional imaging (Lin et al., 2018). In a study with children, Cao and colleagues (2013) reported decreased structural connectivity in ADHD group as compared with NT controls, which was mostly observed in a prefrontal network and in its connections with the parietal and somatomotor areas. Connectivity strength in this network was further associated with inattention symptoms. In another child study, Hong and colleagues (2014) found in children and adolescents with ADHD, as compared with NT controls, decreased structural connectivity in a widespread network connecting the prefrontal, parietal, and occipital lobes, as well as the basal ganglia and the cerebellum. In conclusion, studies utilizing NBS suggest that ADHD is associated with relatively widespread changes in structural and functional brain connectivity (for functional connectivity studies in children and young adults, see Cocchi et al., 2012; Tao et al., 2017; Zhan et al., 2017).

In graph theoretical analysis, local or global topology of the network is quantified with metrics assumed to reflect information processing in the brain (Bullmore & Sporns, 2009). Graph metrics can be generally divided to those reflecting integration (ability to combine distributed information) or segregation (separated processing of specialized information systems) of the brain networks (Rubinov & Sporns, 2010). Previous research has revealed increased segregation and decreased integration of structural and functional networks, as well as regional (nodal) changes, in children, adolescents, and young adults with ADHD (Beare et al., 2017; Cao et al., 2013; Cocchi et al., 2012; Griffiths et al., 2016; Tao et al., 2017).

DW-MRI studies utilizing graph theory show both global (He et al., 2022; Wang et al., 2021) and local connectivity differences between adults with and without ADHD (He et al., 2022; Sidlauskaite et al., 2015; Wang et al., 2021). Sidlauskaite and colleagues (2015) reported local hypoconnectivity in parietal, temporal, occipital, and cerebellar areas, and local hyperconnectivity particularly in inferior prefrontal, thalamic, parietal, and occipital areas in adults with ADHD. In the temporal and inferior parietal cortex, weaker connectivity was further associated with more severe ADHD symptoms and stronger connectivity in the right putamen was associated with hyperactivity-impulsivity symptoms. Wang and colleagues (2021) reported that adults with ADHD have lower global network efficiency and smaller density of ‘rich-clubs’ than NT controls in several cerebral and subcortical structural hub nodes, both results reflecting decreased integration, a phenomenon mirrored in adult functional connectivity studies (e.g., Fan et al., 2019; Pretus et al., 2019). Opposite to the child studies, He and colleagues (2022) observed decreased segregation in an adult ADHD group, in relation to NT controls, globally in the brain, as well as locally in the left parahippocampal gyrus and right supplementary motor area and ‘modules’ in central and left-sided frontal areas. In functional connectivity studies, both increased (e.g., Fan et al., 2019; Pretus et al., 2019) and decreased (e.g., Lin et al., 2018) segregation in ADHD have been found. Contrary to the studies described above, a study by Hearne and colleagues (2021) found no differences in DW-MRI data between adults with and without ADHD. However, they reported altered structure-function coupling in the frontal-parietal-sensory networks in the adults with ADHD.

Besides small number of DW-MRI studies examining whole-brain networks in adults with ADHD, the methods in these studies have also been limited. For instance, all adult network studies, except the one by Hearne and colleagues (2021), used diffusion tensor imaging tractography, which is unable to reliably estimate complex fiber structures within a voxel, such as crossing, bending, and parting fibers (Tournier et al., 2011). More sophisticated tractography methods help in revealing network differences based on finer anatomical fiber configurations.

In the present study, we explored structural connectivity changes in adults with ADHD. Our goal was to include only ADHD participants with minimal number of comorbid disorders to decrease the ‘noise’ that other symptoms could potentially cause. Structural networks were reconstructed by constrained spherical deconvolution (CSD)–based tractography, which allows more accurate estimations of complex fiber orientations present up to 90% of voxels (Jeurissen et al., 2013) than commonly used diffusion tensor imaging based tractography, resulting in biologically more plausible tract reconstruction (Auriat et al., 2015; Jeurissen et al., 2011; Reijmer et al., 2012). We then determined whether NBS is able to detect subnetworks in DW-MRI data distinguishing adults with and without ADHD (Zalesky et al., 2010). In addition, we examined group differences by computing graph theoretical metrics for the global and local effects to characterize the abnormal topological brain wiring structure (Bullmore & Sporns, 2009; Rubinov & Sporns, 2010). The combination of these two methods was expected to provide a broad picture of large-scale structural abnormalities, and to capture the integration and segregation of the brain networks. In addition to group differences, we studied associations between structural connectivity and ADHD symptoms, as well as task-based attention measures. Finally, due to the discrepant findings in the previous DW-MRI studies related to ADHD (see, e.g., He et al., 2022; Hearne et al., 2021; Sidlauskaite et al., 2015), we used two different brain parcellations to verify the results.

On the basis of previous studies reporting local and edge-level white matter changes of ADHD adults, we expected that NBS would find widespread differences between adults with ADHD and NT controls. In graph theoretical analysis, we assumed to find differences in both segregation and integration, as has been the case in many previous structural and functional connectivity studies in children and adults. However, since there are still only a few network studies using DW-MRI in adults with ADHD, we did not make any assumptions about the direction of these effects.

## RESULTS

### Behavioral Characteristics

**Figure 1.**
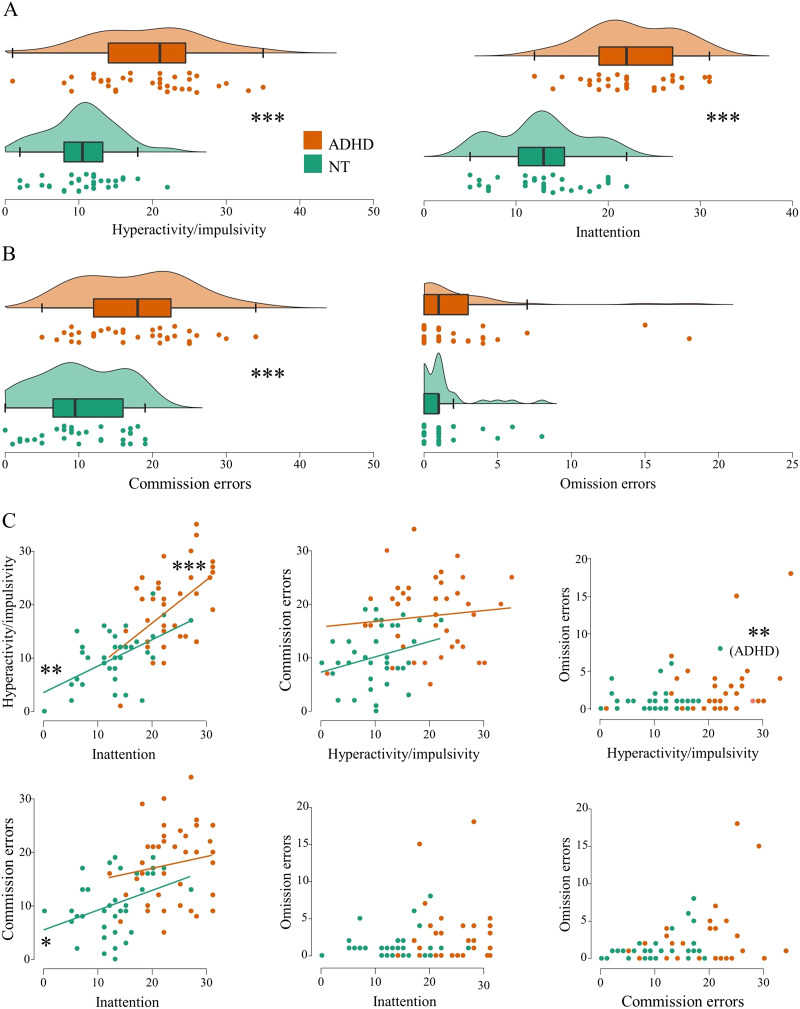
(A) ADHD symptoms and (B) number of errors in CPT-2 displayed by raincloud plots showing individual data points, density plots and box plots by group. (C) Scatter plots and regression lines of associations between ADHD symptoms and number of errors in CPT-2. Regression lines are not added to plots including omission errors, because their distributions within groups do not follow a normal distribution. ***p < .001, **p < .01, *p < .05.

The groups did not differ in terms of age, gender, handedness, general cognitive abilities, education, mood, or in alcohol consumption (Table 1 and Supporting Information Figure S1). ADHD group reported more inattention and hyperactivity-impulsivity symptoms than neurotypical adults and did more commission errors in the continuous performance test (Table 1 and Figure 1). In the ADHD group, inattention was positively correlated with hyperactivity-impulsivity symptoms (*r*(38) = .53, *p* < .001), and hyperactivity-impulsivity symptoms were positively correlated with the amount of omission errors in CPT-2 (*r*_*s*_(38) = .45, *p* = .004). In the NT group, inattention was positively correlated with hyperactivity-impulsivity symptoms (*r*(34) = .52, *p* = .001) and the amount of commission errors in CPT-2 (*r*(34) = .37, *p* = .025). Otherwise, there were no statistically significant correlations between ADHD symptoms and CPT-2 errors.

**Table 1.** Demographic and clinical characteristics of the participants

Characteristics	ADHD (*N* = 40)	NT (*N* = 36)	Test statistic (*df*)	*p*
Age, *M* (*SD*)	28.35 (5.13)	28.42 (7.81)	*t* (59.38) = 0.043	.97
Gender, male/female	17/23	14/22	χ^2^ (1) = 0.10	.75
Handedness, right/left	34/6	33/3	χ^2^ (1) = 0.81	.37
General cognitive abilities				
Vocabulary, *M* (*SD*)	11.38 (2.54)	11.11 (2.44)	*t* (74) = −0.46	.65
Matrix reasoning, *M* (*SD*)	12.43 (2.82)	13.17 (2.22)	*t* (74) = 1.26	.21
Education*	6/11/7/1/5/5	3/12/2/1/5/9	χ^2^ (5) = 4.84	.44
Mood, *M* (*SD*)	5.15 (3.42)	5.00 (3.67)	*t* (73) = −0.18	.86
Alcohol consumption, *M* (*SD*)	4.30 (2.39)	3.57 (2.03)	*t* (73) = −1.41	.16
ADHD symptoms (ASRS)				
Inattention, *M* (*SD*)	23.36 (5.08)	12.78 (5.54)	*t* (74) = −8.69	< .001
Hyperactivity-impulsivity, *M* (*SD*)	19.58 (7.27)	10.19 (4.96)	*t* (69.15) = −6.63	< .001
CPT errors (CPT-2)				
Omission errors, *Mdn* (*IQR*)	1 (0–3)	1 (0–1)	*U* = 670	.59
Commission errors, *M* (*SD*)	17.77 (7.01)	10.22 (5.48)	*t* (74) = −5.19	< .001

*Note*. *df* = degrees of freedom, *M* = mean, *SD* = standard deviation, ASRS = Adult ADHD Self-Report Scale, CPT-2 = Conners Continuous Performance Test 2 *Mdn* = median, *IQR* = interquartile range.

* Education scale in order of appearance: comprehensive school/upper secondary school/vocational school/community college level/bachelor’s degree/master’s degree.

### NBS: Group Differences

**Figure 2.**
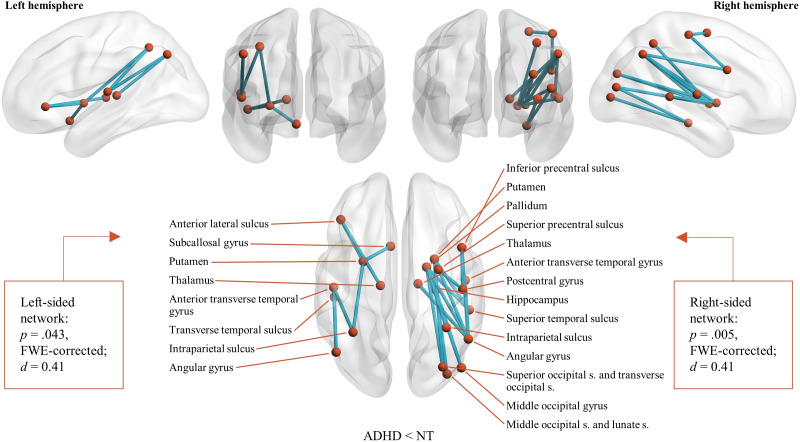
Networks identified with NBS differentiating adults with and without ADHD. The ADHD group showed decreased connectivity compared to the NT group in intrahemispheric networks connecting multiple subcortical and cortical structures.

**Figure 3.**
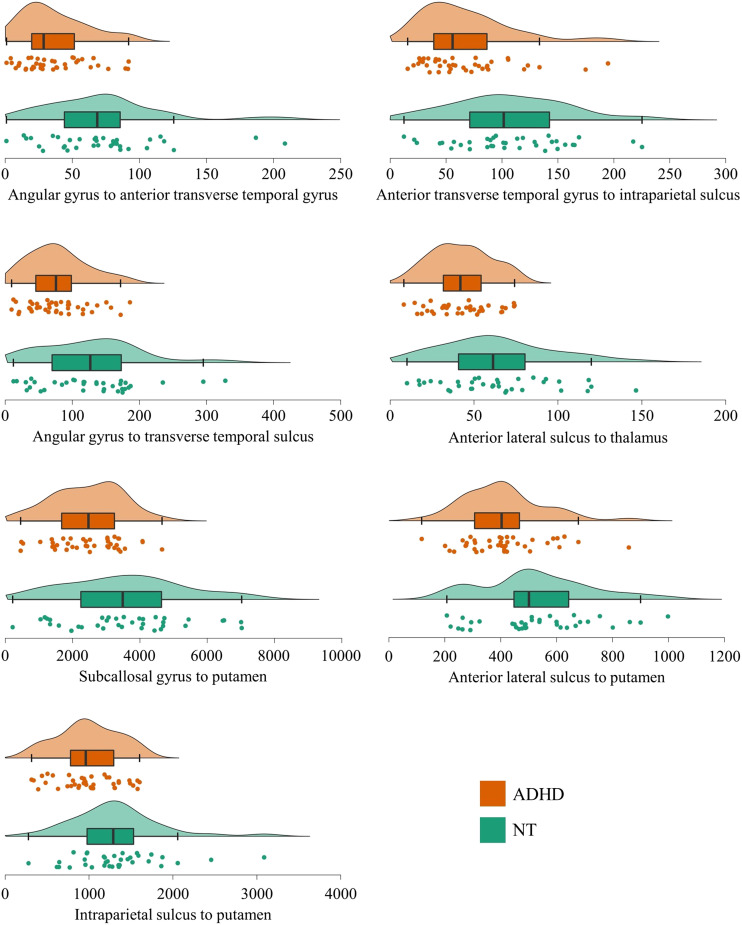
Raincloud plots displaying individual data points, density plots, and box plots by group of the edge weight distributions in the left-sided networks differentiating adults with and without ADHD.

**Figure 4.**
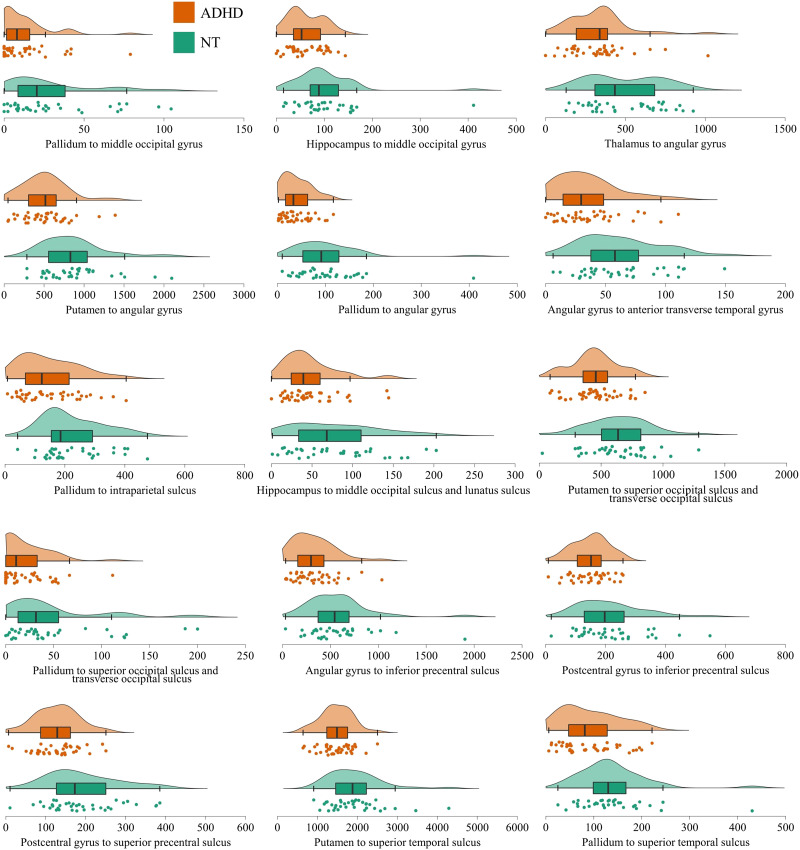
Raincloud plots displaying individual data points, density plots, and box plots by group of the edge weight distributions in the right-sided networks differentiating adults with and without ADHD.

We found decreased connectivity in the ADHD group compared to the neurotypical group in networks connecting multiple subcortical and cerebrocortical areas (Figure 2, Figure 3, Figure 4, and Supporting Information Figure S2 and Tables S1 and S2). The subcortical areas included the thalamus and parts of the striatum, basal ganglia, and limbic system. Cortical regions encompassed various areas in the occipital, parietal, temporal, and frontal lobes. With a *t*-statistic threshold 3.0, two unilateral networks were identified: one on the left and one on the right side of the brain. The networks shared areas in the parietal and temporal lobes, as well as subcortical structures. However, occipital areas were present only in the right-sided network. Two unilateral networks were identified also with a *t*-statistic threshold of 3.5, but the extent of the networks was smaller. When intensity was used as a measure of network size, both networks were found also with a *t*-statistic threshold 4.0, but by using extent as network size measure, the left-sided network was no longer present with the more stringent threshold. Otherwise, the networks were identical with either extent or intensity. No statistically significant networks with increased connectivity in the ADHD group compared to the neurotypical group were identified with any *t*-statistic threshold either with extent (smallest *p* value: *t* = 3.0, *p* = .45, FWE-corrected) or intensity (smallest *p* value: *t* = 3.0, *p* = .30, FWE-corrected) as a measure of network size.

To ensure that the results were not significantly affected by outliers in the NT group (see Figures 3 and 4), we reran the NBS analysis after removing all NT participants whose edge weight exceeded 2 *SD* from the group mean for two or more connections in the networks differentiating the participants with and without ADHD (six participants in total). The results were replicated with only minor changes in the networks (see Supporting Information Figure S3).

### NBS: Associations With Behavioral Measures

In the networks differentiating adults with and without ADHD (see above), the mean connectivity (mean of edge weights) did not correlate with either ADHD symptoms or performance in CPT-2 within either of the groups examined separately.

**Figure 5.**
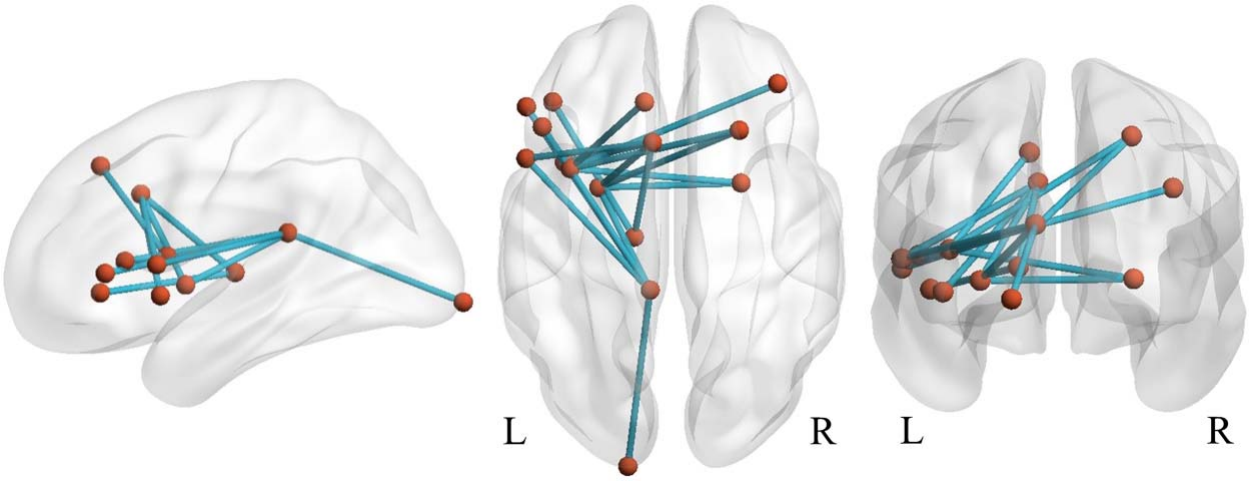
The network identified with NBS representing hypoconnectivity associated with higher amount of commission errors in CPT-2 across all participants (*p* = .005, FWE-corrected; *d* = 0.37). L = left hemisphere, R = right hemisphere.

**Figure 6.**
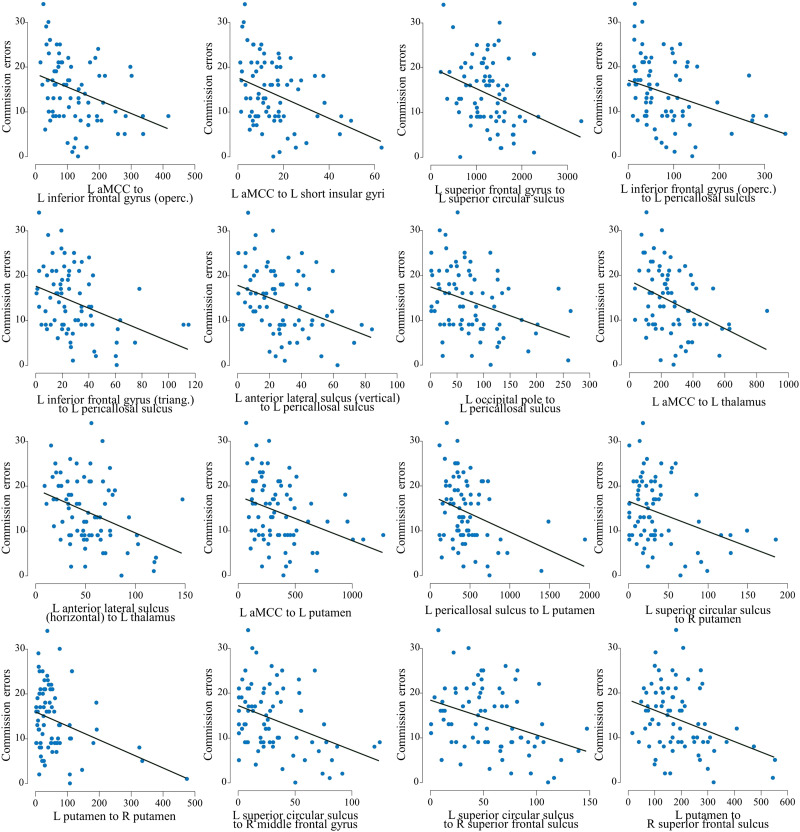
Scatter plots and regression lines of associations between the number of commission errors in CPT-2 and edge weights of the network representing hypoconnectivity associated with higher amount of commission errors in CPT-2 across all participants. L = left, R = right, aMCC = middle-anterior part of the cingulate gyrus and sulcus, operc. = opercular part, triang. = triangular part.

The NBS analysis for the associations between behavioral measures and edge weights across all participants identified a network in which edge weights were negatively correlated with commission errors in CPT-2 (Figure 5 and Figure 6 and Supporting Information Table S1). This network included the left thalamus, putamen bilaterally, and frontal corticocortical structures predominantly in the left hemisphere. The network was not present with *t*-statistic thresholds 3.5 and 4.0 (smallest *p* values: *t* = 3.5, *p* = .37; *t* = 4.0, *p* = 1). No networks associated with other behavioral measures were found (smallest *p* values: ASRS inattention, *t* = 4.0, *p* = .07; ASRS hyperactivity-impulsivity, *t* = 3.5, *p* = .34; CPT-2 omission errors, *t* = 4.0, *p* = .32).

### Graph Theoretical Analysis

In the global analyses, we did not find significant differences between ADHD and control groups in any studied metrics (all *p* > .05; Supporting Information Table S3). Increased rich-club organization was found in subjects with ADHD for degree thresholds from 70–71, 79–80, 82–90, and 92 (*p* < 0.05). However, the average rich-club coefficient was not statistically different between the groups (*p* = 0.055), and the degree-specific results did not endure multiple correction for the number of degree thresholds used. In the local analyses, the strength of the left temporal pole was decreased in the ADHD group with a statistical significance level of *α* = 0.001 (*F*(1, 76) = 13.8, *p* < .001). However, the local result did not survive Bonferroni correction accounting for the total number of nodes. A positive correlation between hyperactivity-impulsivity symptoms and normalized modularity within the ADHD group was found (*r*(38) = .33, *p* = .04), but this result did not survive Bonferroni correction accounting for the number of tests performed. No other correlations between the ADHD symptoms or performance in CPT-2 and graph theory metrics were found within either of the groups examined separately or by conducting the analysis across all participants (Supporting Information Table S4).

### Comparative Analyses With the Schaefer Parcellation

The NBS analysis with the Schaefer parcellation revealed hypoconnectivity in the ADHD participants compared to the NT controls in a right-sided network, which largely overlapped with the network identified using the Destrieux parcellation (Supporting Information Figure S4 and Table S5). All results from the graph theory analyses were replicated with the Schaefer parcellation apart from the correlation between hyperactivity-impulsivity symptoms and normalized modularity within the ADHD group (Supporting Information Tables S6 and S7).

## DISCUSSION

Prior DW-MRI literature in adults with ADHD has been quite limited. This field has been especially lacking studies with elaborated methods that are able to pinpoint fine-grained structural abnormalities in ADHD (e.g., CSD) and account for various confounding factors, in particular possible group differences in participants’ motion (see Aoki et al., 2018). We report comprehensive DW-MRI analyses in an adult ADHD sample with two relatively understudied methods (NBS and graph theory), employing a homogenous sample of ADHD participants with no major comorbid disorders. We also confirmed that our participants with ADHD did not move in the scanner more than NT controls.

We first identified group differences in structural subnetworks with NBS. This analysis revealed edge-level hypoconnectivity in participants with ADHD in unilateral left- and right-sided networks encompassing several subcortical and cerebrocortical structures (see Figure 2). We also identified a predominantly left-frontal network in which hypoconnectivity was associated with a greater amount of commission errors in CPT-2 across all participants (see Figure 5). Graph theoretical analysis characterizing the topological organization of the white matter pathways did not show any global differences between ADHD adults and NT controls. However, we observed decreased strength of the left temporal pole in the ADHD group.

### Network-Based Statistics

Our NBS results agree with several prior studies reporting structural or functional hypoconnectivity in ADHD. Hong and colleagues (2014) identified hypoconnectivity in children with ADHD as compared to neurotypical controls in a widespread network, which shared many cortical and subcortical areas with the networks identified in the present study. Decreased connectivity in children with ADHD has also been reported in structural networks between prefrontal, parietal, and somatomotor areas (Cao et al., 2013), and in functional studies (Tao et al., 2017; Zhan et al., 2017). However, in adults with ADHD, hyperconnectivity is more frequently observed than hypoconnectivity. In a recent study, He and colleagues (2022) found higher structural connectivity between subcortical and several cerebrocortical areas in adults with ADHD than in NT controls. Increased connectivity in adults with ADHD was also revealed in a functional study by Lin and colleagues (2018). Although there were few overlapping nodes between the networks identified in these studies and the one observed in the present study, there were also considerable differences. Importantly, however, the structural study of Hearne and colleagues (2021) utilizing advanced tractography methods, as we did, found no networks distinguishing adults with or without ADHD. Hearne and colleagues pointed out that head movements can lead to false positive findings in DW-MRI studies, and argued that careful controlling of in-scanner movements in their study could explain why they observed no group differences, even though such results have been described in the previous studies. In the present study, however, altered connectivity was found in the ADHD group even though there were no between-group motion differences. Although both their and our study tried to minimize neurodevelopmental, psychiatric, and neurological comorbidity, different results could still reflect overall heterogeneity of ADHD symptoms.

The networks identified in the present study mainly represent weaker connections between areas of the dorsal and ventral attention networks (Vossel et al., 2014), somatomotor and visual areas, and subcortical structures, including parts of the striatum and basal ganglia. This could be manifested, for example, in reduced integration of sensory processing and aberrant top-down attentional control of the related sensory systems in ADHD. These same systems have been associated with ADHD in previous research: Meta-analyses have found evidence for aberrant connectivity between the ventral attention and somatosensory networks and the fronto-parietal network (Gao et al., 2019) and disrupted activation in visual and dorsal attention networks in adults with ADHD and in ventral attention and somatomotor networks in children with ADHD (Cortese et al., 2012). In addition, the striatum is thought to play an important role in ADHD symptomatology (Castellanos & Proal, 2012; Cubillo et al., 2012). While the previous NBS studies shared some areas with our study, their findings focused on decreased segregation of the default and salience networks from other networks (Lin et al., 2018), and between areas encoding emotional and visual processing and the default mode network (He et al., 2022). Therefore, the results of the prior adult ADHD studies may represent different aspects of alterations in the brain network wiring.

Although the networks differentiating adults with and without ADHD were not associated with behavioral measures within either group, we found a network in which hypoconnectivity was related to higher number of commission errors across all participants. Much like in the present results, an impulsivity factor comprised of commission errors among other measures of CPT was linked to reduced microstructural properties in occipital, frontal, and striatal areas, and the thalamus in a previous population-based study (Gagnon et al., 2023). Similarly, Hong and colleagues (2014) found that commission errors in the CPT were associated with reduced fractional anisotropy in some connections of a widespread network that distinguished children with ADHD from controls in their study. However, differences in the CPT tasks and age cohorts across studies make direct comparisons between the previous studies and the present study challenging. The brain network associated with CPT-2 in the present study, which included parts of fronto-parietal and salience networks (Uddin et al., 2019), may be viewed as associated with sustained attention independent of the diagnostic status.

In the present study, omission errors in CPT-2 were associated with hyperactivity-impulsivity in the ADHD group, and CPT-2 commission errors with inattention in the NT adults. This could be regarded as surprising, giving that in children with ADHD commission errors are thought to reflect hyperactivity-impulsivity and omission errors inattention (but see, e.g., Epstein et al., 2003). However, in adults the results have been mixed. In a recent review, Pagán and colleagues (2023) reported that in adult ADHD populations, CPT omission errors are actually typically associated with hyperactivity-impulsivity, and CPT commission errors with both hyperactivity-impulsivity and inattention. The present findings are largely consistent with this recent review. However, in adults the subtyping of ADHD according to hyperactivity-impulsivity and inattention subscales is overall less reliable (e.g., Gibbins et al., 2010; Willcutt et al., 2012).

While the present NBS analysis focused on edges of the connections, most of the previous studies have examined local white matter changes in ADHD (Bode et al., 2015; Chaim et al., 2014; Cortese et al., 2013; Konrad et al., 2010; Onnink et al., 2015; see Aoki et al., 2018; Chen et al., 2016; and van Ewijk et al., 2012; for meta-analyses). Although these are two different levels of network characteristics, we would like to note that some pathways that in previous studies have shown consistent differences at local level between participants with and without ADHD (for meta-analyses, see Aoki et al., 2018; Chen et al., 2016; van Ewijk et al., 2012) overlap with the network that showed group differences at edge level in the present study. More specifically, similar to the previous studies, the present NBS analysis revealed aberrant white matter pathways connecting frontal, temporal, parietal, and occipital areas. By measuring the number of streamlines instead of regional fractional anisotropy values, we provided measures that are assumed to capture the actual connectivity more accurately (Huang & Ding, 2016; Yeh et al., 2016).

As can be seen from the comparison with NBS studies above, our results agree with the hypoconnectivity findings reported in child ADHD populations (Cao et al., 2013; Hong et al., 2014). In studies observing the microstructural properties of white matter bundles in children, abnormalities have mostly been seen in the superior longitudinal fasciculus, cingulum, and thalamic radiations, structures that connect the same areas that appear in our NBS networks (Connaughton et al., 2022). Similarly, a recent study found that attention and impulsivity were associated with reduced microstructural properties in the occipital and temporal cortices, somatomotor network, dorsal striatum, and thalamus in a normative child population (Gagnon et al., 2023). However, in children with ADHD, differences in the frontostriatal connections, corpus callosum, and corona radiata have also been reported (Connaughton et al., 2022), as well as abnormalities in corticospinal and corticopontine tracts and the uncinate fasciculus by using a novel fixel-based method (Fuelscher et al., 2021). Furthermore, prefrontal cortex and the cerebellum have been well represented in connectomic studies in child ADHD populations (Cao et al., 2013; Hong et al., 2014).

Previous studies have also revealed that the symptom characteristics are quite different in children and adults with ADHD. Especially hyperactivity-impulsivity decreases in the course of development, in tandem with diminishing abnormalities in brain structure and function (Frodl & Skokauskas, 2012; Kasparek et al., 2015; Nakao et al., 2011; Rubia et al., 2014). However, some studies have found qualitatively distinct functional connectivity aberrancies in children and adults with ADHD (Guo et al., 2020; Liu et al., 2023), implicating pathophysiological changes during development beyond diminishing brain alterations. Longitudinal studies are needed to further clarify the nature of developmental connectivity patterns in ADHD. It is also possible that changes in structural brain connectivity are more local in children than in adults and become sparser with the development (Dennis et al., 2013; Hagmann et al., 2010). To further examine these sparse effects in topological organization of the networks rather than number of streamlines, we conducted a graph analysis that goes beyond the local connection strength. The results of these analyses are discussed below.

### Graph Theoretical Analysis

Although graph theory is becoming an increasingly common analytical approach in brain imaging, there are only few previously published structural MRI studies utilizing this approach in adults with ADHD and studying whole-brain topological features (He et al., 2022; Hearne et al., 2021; Sidlauskaite et al., 2015; Wang et al., 2021). While some previous studies have found global differences in adults with and without ADHD (He et al., 2022; Wang et al., 2021), others have come to a conclusion that ADHD-related alterations are not seen in global topology (Hearne et al., 2021; Sidlauskaite et al., 2015). While the results of the present study bring more evidence to the latter, more studies are needed to demonstrate if there are more subtle subnetworks that characterize aberrant structural connectivity in adult ADHD.

Adults with ADHD presented increased rich-club organization compared to the NT participants, which is in contrast to the findings of decreased rich-club density in adults with ADHD in the study by Wang and colleagues (2021). In the local graph theoretical analyses, the left temporal pole showed decreased strength in the participants with ADHD (*p* < .001). However, as the present results regarding rich-club organization or local graph theoretical metrics did not survive multiple testing correction, we refrain from interpreting these findings with greater detail.

Correlation analyses performed within the ADHD group revealed that higher modularity, reflecting increased segregation of separate information systems, was related to more severe hyperactivity-impulsivity symptoms within the ADHD group. However, this result did not survive the multiple testing correction either. Otherwise, the analyses did not show clear links between the graph metrics and symptoms or CPT-2 performance. We used a common CPT variant shown to successfully capture some of the core symptoms of ADHD (Pagán et al., 2023). However, the lack of prior studies examining associations between brain topology and CPT performance makes it difficult to evaluate if the type of CPT employed in the study could affect the results. Nevertheless, our results are generally in line with previous research suggesting that only local network properties are associated with ADHD symptoms (Hilger & Fiebach, 2019; Sidlauskaite et al., 2015). In this study, however, we did not find significant correlations with the local properties, possibly due to limited power resulting from the relatively low sample size.

As in studies using NBS, most of these studies have used diffusion tensor imaging (DTI) tractography. Previous results may have been contaminated also by other various sources of bias is streamlines tractography, such as seeding (Girard et al., 2014), invalid streamlines (Smith et al., 2012), uncorrected streamline density (Smith et al., 2013, 2015), and varying intracranial volume (Klein et al., 2019). These potential biases have been properly corrected in this study in contrast to most previous studies. The only other study using advanced tractography methods (Hearne et al., 2021) also found no results on either global or local level, emphasizing the need for state-of-the-art methods to be selected. However, unlike Hearne and colleagues (2021) we observed with NBS group differences in edge level (see above). In our study, the groups did not differ in amount of motion during scanning and were without major psychiatric, neurological, or neurodevelopmental comorbidities. These common problems potentially affecting the results in ADHD research can explain the differences between previous studies and our results at least to some degree.

There could be several reasons why ADHD-related alterations were observed in the NBS analysis, although the graph theoretical analysis did not reveal any group differences. The most obvious explanation is that NBS may be more sensitive to reveal edge-level differences, as its method is developed to optimally account for the multiple comparison problem (Zalesky et al., 2010). It is, however, difficult to directly compare the two methods as there are differences in how the statistics are performed in them: NBS focuses on edge-level information, forming interconnected subnetworks based on associations with variables of interest or group differences (Zalesky et al., 2010). Graph theoretical analysis, in turn, describes nodal properties defined by their connectivity to all other areas on the brain, or global whole-brain features based on attributes of the whole connectome (Bullmore & Sporns, 2009). Other explanation could be that, in general, alterations in the structural brain networks in ADHD diminish with increasing age (Frodl & Skokauskas, 2012; Kasparek et al., 2015; Nakao et al., 2011; Rubia et al., 2014) and thus, it is possible that ADHD-related differences could be too subtle to detect when considering whole-brain topological features, or that local node-level differences are too small to survive correction for multiple analysis.

It is possible that in the long run, diffusion imaging could help in specifying the ADHD diagnoses. In many cases, MRI is collected to rule out other possible alternative explanations in the clinical evaluation. Finding reliable structural brain markers is still underway. The present findings of robust group differences already in relatively modest sample size hold a promise that such methods might be possible in the future.

### Limitations of the Present Study

Typical limitations in brain imaging research of adult ADHD include the potential influence of medication, participants’ motion, heterogeneity of the patient sample, sufficient sample size, and choices made in the statistical testing. Like in most of the DW-MRI studies in adults with ADHD (see Aoki et al., 2018), almost all present participants with ADHD regularly used stimulant medication (however, see Hearne et al., 2021). Long-term use of such medication is shown to decrease abnormalities in brain structure and function in participants with ADHD (Frodl & Skokauskas, 2012; Hart et al., 2013; Nakao et al., 2011). Thus, it is possible that effect sizes were relatively small at least partially due to the medication. In addition, even though participants had a 24-h washout period from psychostimulants before coming to the experiment, possible effects of pharmaceutical treatment on attention task performance or self-reported symptoms cannot be ruled out. Group differences in participant motion, in turn, may lead to overestimation of abnormal structural connectivity. In our case, no group differences in participant motion were found. We made an effort to obtain a homogeneous sample so that the participants with ADHD would not have any major comorbid disorders confounding the results. Compared with previous studies, the group sizes were about average. It should be also noted that the present DW-MRI data acquisition may be considered suboptimal due to the low diffusion weighting (Tournier et al., 2013) and the lack of reverse-phase encoding that could be used to correct for EPI-induced distortions (Andersson et al., 2003; Irfanoglu et al., 2012).

### Conclusions

The goal of the present study was to delineate abnormal wiring of the structural connectome in adults with ADHD. Prior to this study, only a few related studies in the adult ADHD population have been published. We found hypoconnectivity in ADHD participants in two networks covering areas related to attentional control and sensory processing. Furthermore, our graph theoretical analysis characterizing the topological organization of the white matter pathways revealed no group differences between the adults with ADHD and the NT controls. In summary, our results suggest that abnormal wiring of the brain in adult ADHD is manifested as a local hypoconnectivity reflecting insufficient integration of sensory processing from different modalities and attentional control over them.

## MATERIALS AND METHODS

### Participants

Forty individuals with ADHD and 36 neurotypical controls participated in this study (see Table 1). The participants with ADHD were recruited at the Neuropsychiatry outpatient clinic of the Helsinki University Hospital and at two private clinics in the Helsinki metropolitan area (Diacor Healthcare Services in the city of Helsinki and ProNeuron in the city of Espoo). All patients were prescreened at the clinic. The psychiatrists recruiting the participants with ADHD used the Structured Clinical Interview for DSM-IV Axis I Disorders (SCID-I) and the Mini-International Neuropsychiatric Interview (M.I.N.I.) to exclude comorbid disorders as part of their regular clinical assessment. The participants were excluded if they had any other severe psychiatric or neurological disorders than ADHD, including head trauma demanding treatment, substance abuse, or other addictions. The NT controls were recruited mainly via email lists at vocational schools, adult high schools, polytechnics, and universities, and via personal contacts of the authors. In both groups, participants had to be native Finnish speakers, have normal or corrected-to-normal vision, sufficient hearing, and meet the eligibility criteria for MRI. The study was reviewed and approved by the Ethics Committee for Gynecology and Obstetrics, Pediatrics and Psychiatry of the Helsinki and Uusimaa Hospital District. All participants gave their informed consent according to the Declaration of Helsinki. The participants were reimbursed with €60 if they participated only to the first MRI measurement and €240 if they continued to a cognitive intervention that is reported in a separate manuscript (Salmi et al., 2020).

ADHD was diagnosed according to the Diagnostic and Statistical Manual of Mental Disorders, Fourth Edition (DSM-IV). In addition to the original diagnostic interview, we conducted the Conners’ Adult ADHD Diagnostic Interview for DSM-IV to confirm the participants’ current status (Epstein et al., 2001). The patients met criteria for either only inattention or both inattention and hyperactivity. Of the included participants, four had migraine, two had hypothyroidism, and two had experienced mild epilepsia symptoms in childhood but with no treatment needed since that time. In addition to 33 participants with ADHD using stimulants, in total four participants had been prescribed medicine for migraine, one for mild depression (selective serotonin reuptake inhibitor), and two for hypothyroidism. Participants had a 24-h washout period from psychostimulants before coming to the experiment. Matrix reasoning and Vocabulary tests of the Wechsler Adult Intelligence Scale (WAIS-III, Wechsler, 2005) were conducted to assess the general cognitive abilities.

### Self-Ratings

Adult ADHD Self-Report Scale (ASRS) version 1.1 was used to self-rate the ADHD symptoms (Kessler et al., 2005), mood was assessed with the Depression Scale (Salokangas et al., 1995), and alcohol consumption was assessed with the Alcohol Use Disorders Identification Test–Consumption (Bush et al., 1998).

### Continuous Performance Test (CPT)

For CPT (Rosvold et al., 1956), we used the version available in the Psychology Experiment Building Language (PEBL) toolbox (Mueller & Piper, 2014), which is a faithful implementation of the Conners Continuous Performance Test 2 (Conners & MHS Staff, 2000). The participants were presented with a sequence of letters with fixed alternating intervals (1,000 ms, 2,000 ms, and 4,000 ms). They were required to press the space bar for each letter, except for the letter X (probability of occurrence 9.7%). Two dependent variables were used: Omission errors (inattention) and Commission errors (impulsivity). There were 360 trials, and the duration of the task was approximately 14 minutes.

### MRI Acquisition

We collected DW-MRI data at Advanced Magnetic Imaging Centre (Aalto University) using a Siemens MAGNETOM Skyra 3 T scanner (Siemens Healthcare, Erlangen, Germany), which was mounted with a 30-channel head coil. Diffusion-weighted (DW) images were acquired using echoplanar imaging (EPI) in 64 different gradient directions (*b* = 1,000 s/mm^2^) and additional 10 unweighted scans (*b* = 0 s/mm^2^) were acquired. Echo time (TE) was 80 ms, repetition time (TR) 9,000 ms, resolution 2.5 mm × 2.5 mm × 2.5 mm and field of view (FOV) 240 mm × 240 mm. Total of 70 axial slices were taken. Imaging time per participant was approximately 11 minutes. T1-weighted 3D anatomical images were acquired using a magnetization prepared rapid gradient echo sequence (MPRAGE) with following parameters: TE = 3.3 ms, TR = 2,530 ms, inversion time = 1,100 ms, resolution 1 mm × 1 mm × 1 mm, FOV 256 mm × 256 mm and flip angle 7°. Total of 176 sagittal slices were taken. Imaging time per participant was approximately 6 minutes.

### Preprocessing of Imaging Data

Preprocessing of the DW images, tractography, and network reconstruction were performed using FMRIB Software Library (FSL) (Jenkinson et al., 2012) and MRtrix3 (Tournier et al., 2019). First, the data were corrected for participant motion and eddy current induced distortions using FSL’s eddy (Andersson & Sotiropoulos, 2016) and for EPI-induced distortions by using nonlinear registration to T1 images (Irfanoglu et al., 2012). Participants’ movement during DW imaging was quantified with root-mean-square (RMS) movement and restricted RMS movement. Groups did not differ from each other in any movement parameter [Wilk’s *λ* = 0,89, *F*(4, 71) = 2.21, *p* = .08, partial *η*^2^ = .11; Supporting Information Table S8]. Also, the maximum motion values were divided relatively evenly across the two groups (Supporting Information Table S8).

### Tractography

For tractography, constrained spherical deconvolution (CSD) was used (Tournier et al., 2007). With CSD, multiple fiber orientations within a single voxel can be estimated by calculating fiber orientation distributions (FOD) using rotational and spherical harmonics (Tournier et al., 2007). This way complex fiber configurations, for instance, crossing, bending, and parting fibers, present in up to 90% of the voxels (Jeurissen et al., 2013), can be estimated more accurately than with traditionally used DTI, even with low b-values (Auriat et al., 2015; Reijmer et al., 2012). CSD has also good sensitivity and specificity compared to other higher-level tractography methods (Wilkins et al., 2015). Whole-brain streamlines tractography by using up to sixth-order spherical harmonics and the iFOD2 algorithm (Tournier et al., 2010) was used to reconstruct 10 million streamlines for each subject. Streamlines were seeded from the interface between the cortical gray matter and white matter, which reduces the overestimation of fiber densities in long connections (Li et al., 2012; Smith et al., 2012), and their anatomical feasibility was improved by using anatomically constrained tractography (Smith et al., 2012). The density of the reconstructed streamlines was corrected to match more closely the underlying FODs by using spherical deconvolution informed weighting of tractograms (SIFT2; Smith et al., 2013, 2015).

### Construction of the Structural Brain Connectivity Networks

The brain was automatically parcellated to cortical and subcortical regions using the Destrieux atlas (Destrieux et al., 2010) in the FreeSurfer image analysis suite (https://surfer.nmr.mgh.harvard.edu). Subcortical structures in FreeSurfer segmentation were then replaced using FIRST algorithm (Patenaude et al., 2011) of the FSL toolbox (Smith et al., 2004). This resulted in total of 164 gray matter areas representing the nodes in the network. Connections, or edges, between the nodes were then constructed using the streamlines (estimated white matter trajectories) detected by the tractography algorithm (Jeurissen et al., 2011). Two nodes were set to be connected by an edge when one or more streamlines ended in both nodes. We used weighted edges in all analyses. Edges were weighted by using the streamline count shown to be the most reproducible weight (Roine et al., 2019). To reduce the number of spurious connections, networks were thresholded by removing connections that had less than 10 streamlines on average across all subjects. Due to seeding from the gray matter–white matter interface, consistency-based thresholding proposed by Roberts and colleagues (2017) was not needed to correct for the seeding bias of longer connections.

There were no obvious differences in the layout of edge weights between participants (Supporting Information Figure S5). One participant in the ADHD group had a significantly higher total count of streamlines than the rest of the participants (see Supporting Information Figure S6). However, removing this outlier participant from the analyses did not considerably affect the results (see Supporting Information Figure S7 for minor changes in the NBS networks).

### Statistical Analyses

Normality of scalar demographic and clinical characteristics was tested with the Shapiro-Wilk test and by visually inspecting the Q-Q plots. Number of omission errors was not normally distributed and was therefore analyzed with nonparametric tests: group differences with the Mann-Whitney *U* test and correlations with Spearman’s *ρ*. Other scalar characteristics (age, Vocabulary, Matrix reasoning, mood, alcohol consumption, inattention, hyperactivity-impulsivity, and commission errors) were determined to be normally distributed and were analyzed with parametric tests: group differences with independent samples *t* test and correlations with Pearson’s *r*. However, because of slight skewness in some measures causing rejection of normality in the Shapiro-Wilk test (age, Vocabulary, Matrix reasoning, mood, and alcohol consumption), group differences were double-checked with a nonparametric Mann-Whitney *U* test, but the results remained unaffected. Group differences in gender, handedness, and education were analyzed with *χ*^2^ test for association.

NBS (Zalesky et al., 2010) was used to identify subnetworks in which statistically significant group differences in structural connectivity were present. First, group difference in edge weight was computed between all pairs of nodes [*N*(*N* − 1) / 2 = 13,366] using a two-sample one-tail *t* test. Group differences were measured in both directions (ADHD < NT and ADHD > NT). As the choice of the primary *t*-statistic threshold is somewhat arbitrary, we used multiple thresholds to identify both subtle but extended effects (liberal thresholds) and stronger, more focal differences (conservative thresholds). We started from relatively liberal 3.0 (corresponding to one-tailed *p* = .0018) to match our analysis with the study by Hearne and colleagues (2021), who used the same threshold. Other chosen thresholds were 3.5 (corresponding to one-tailed *p* = .0004), also commonly used in NBS studies (e.g., Cocchi et al., 2012; Lin et al., 2018), and finally, 4.0 (corresponding to one-tailed *p* = .00007) to investigate the robustness of the effect with a fairly conservative threshold. Next, subnetworks were constructed of interconnected edges that exceeded a chosen threshold. The size of each identified subnetwork was then computed by two methods: measuring the extent (number of edges) and intensity (sum of test statistic values across edges) of the subnetwork. We decided to use both measures for network size, as they can reveal different aspects of network-level differences. Extent can better identify distributed subnetworks, and intensity is more sensitive to large effects consisting of few connections (Bullmore et al., 1999). While extent has been commonly used in previous ADHD research (e.g., He et al., 2022; Hearne et al., 2021; Lin et al., 2018), intensity has the advantage of retaining information about the magnitude of the effect (Bullmore et al., 1999). Lack of comparison of the two measures in prior studies make it difficult to determine which one would detect ADHD-related differences more reliably, and thus, no a priori choice was made in the present study. Finally, a family-wise error (FWE) corrected *p* value for each subnetwork was computed using permutation testing. For each of the 10,000 permutations performed, the size of the largest subnetwork was recorded. The corrected *p* value for each subnetwork identified in the actual data was estimated as the proportion of permutations for which a subnetwork of same or greater size was identified. Associations between behavioral measures and edge weights were analyzed with Pearson’s *r*, but otherwise the same procedure was applied.

In the global graph theoretical analyses, we investigated betweenness centrality, normalized clustering coefficient, normalized global efficiency, normalized characteristic path length, normalized modularity, small-worldness, rich-club organization, and strength, while in the local analyses, we investigated node strength, betweenness centrality, local efficiency, and clustering coefficient (Bullmore & Sporns, 2009; Rubinov & Sporns, 2010). *Betweenness centrality* measures the proportion of shortest paths passing through a node, in other words, the importance of a node for the information flow within the network. *Strength* is the number of streamlines originating from a node to any other nodes, and global strength is the average strength of all nodes. *Clustering coefficient* measures the segregation by calculating the number of triangles formed by the node and its neighbors compared to all possible triangles. *Characteristic path length* is the average shortest path length between all possible pairs of nodes. *Global efficiency* is the average inverse shortest path length and is therefore primarily influenced by short paths in contrast to characteristic path length. *Small-worldness* is the fraction of normalized clustering coefficient and normalized characteristic path length, and it illustrates how interconnected the network via shortcut connections is with respect to a lattice network. *Modularity* measures the divisibility of the network into communities with dense intracommunity and sparse intercommunity connectivity. *Rich-club coefficient* measures the extent to which the nodes with a high degree connect to each other in contrast to the other nodes of the network. The coefficient is calculated for varying degree thresholds. The global graph theoretical metrics were normalized with respect to 100 random networks extracted from the degree-, weight-, and strength-preserving null model (Rubinov & Sporns, 2011). Global and local graph theoretical properties between the ADHD group and NT controls were compared with analysis of variance by the general linear model in SPSS 29.0. Associations between graph theoretical properties and ADHD symptoms and CPT-2 performance were analyzed with Pearson’s *r*, except omission errors which were analyzed with Spearman’s *ρ*.

All statistical analyses were performed without covariates to avoid multicollinearity issues, as there were no statistically significant group differences in any background variables.

### Comparative Reliability Analyses

To investigate the reliability of the results and to further match the current analysis to the one by Hearne and colleagues (2021), we repeated all analyses using the seven-network version of the Schaefer parcellation (Schaefer et al., 2018) with 200 parcels. Fourteen subcortical structures segmented with the FIRST algorithm were added to the parcellation as with the Destrieux atlas.

### Brain Visualizations

Brain networks were visualized with the BrainNet Viewer (Xia et al., 2013, https://www.nitrc.org/projects/bnv/).

## DATA AVAILABILITY

The datasets used in this article cannot be publicly shared due to participant privacy and details in the study’s ethical approval. For validation purposes, please contact the corresponding author TT at tuija.tolonen@helsinki.fi for an arrangement of data accessibility.

## SUPPORTING INFORMATION

Supporting information for this article is available at https://doi.org/10.1162/netn_a_00326.

## AUTHOR CONTRIBUTIONS

Tuija Tolonen: Conceptualization; Formal analysis; Funding acquisition; Investigation; Visualization; Writing – original draft; Writing – review & editing. Timo Roine: Formal analysis; Funding acquisition; Writing – original draft; Writing – review & editing. Kimmo Alho: Conceptualization; Funding acquisition; Writing – review & editing. Sami Leppämäki: Conceptualization; Investigation; Resources; Writing – review & editing. Pekka Tani: Conceptualization; Investigation; Resources; Writing – review & editing. Anniina Koski: Investigation; Resources; Writing – review & editing. Matti Laine: Conceptualization; Funding acquisition; Project administration; Writing – review & editing. Juha Salmi: Conceptualization; Data curation; Funding acquisition; Project administration; Supervision; Writing – original draft; Writing – review & editing.

## FUNDING INFORMATION

Matti Laine, Academy of Finland, Award ID: 260276. Matti Laine, Academy of Finland, Award ID: 323251. Kimmo Alho, Academy of Finland, Award ID: 260054. Kimmo Alho, Academy of Finland, Award ID: 297848. Juha Salmi, Academy of Finland, Award ID: 325981. Juha Salmi, Academy of Finland, Award ID: 328954. Matti Laine, Åbo Akademi University Endowment for the BrainTrain project. Timo Roine, Finnish Cultural Foundation. Tuija Tolonen, Vilho, Yrjö, and Kalle Väisälä Foundation of the Finnish Academy of Science and Letters. Open access funded by Helsinki University Library.

## TECHNICAL TERMS

Topological properties: How edges and links are arranged in the network.
Structural brain network: A network formed by the anatomic white matter connections of the brain.
Diffusion-weighted magnetic resonance imaging (DW-MRI): A method to detect water diffusion, that is, displacement of water molecules, to characterize white matter in the brain.
Fractional anisotropy: The directional preference of diffusion. Small values mean equal diffusion to all directions; bigger values mean more diffusion to only one direction.
Graph theoretical analysis: A way to analyze different topological properties of a network.
Network-based statistic: A method to identify subnetworks from whole-brain networks based on how network edge properties are associated with a variable.
Diffusion tensor imaging: A conventional way to model DW-MRI data to characterize white matter properties.
Tractography: Modeling the white matter trajectories.
Constrained spherical deconvolution: Tractography method that allows multiple diffusion orientations to be detected within a single voxel.
Streamline: One estimated white matter trajectory.
