# Supplementary material for: Abnormal wiring of the structural connectome in adults with ADHD
Source: Netw Neurosci. 2023 Dec 22;7(4):1302–25. doi: 10.1162/netn_a_00326 (PMC10631790; doi:10.1162/netn_a_00326)
Supplement: Supplementary file 1 [file netn-7-4-1302-s001.pdf]

Supplementary results

Article title

Abnormal wiring of the structural connectome in adults with ADHD

Authors

Tuija Tolonen, Timo Roine, Kimmo Alho, Sami Leppämäki, Pekka Tani, Anniina Koski, Matti Laine, Juha Salmi

Supplementary figures pp. 2–7
Supplementary tables pp. 8–12

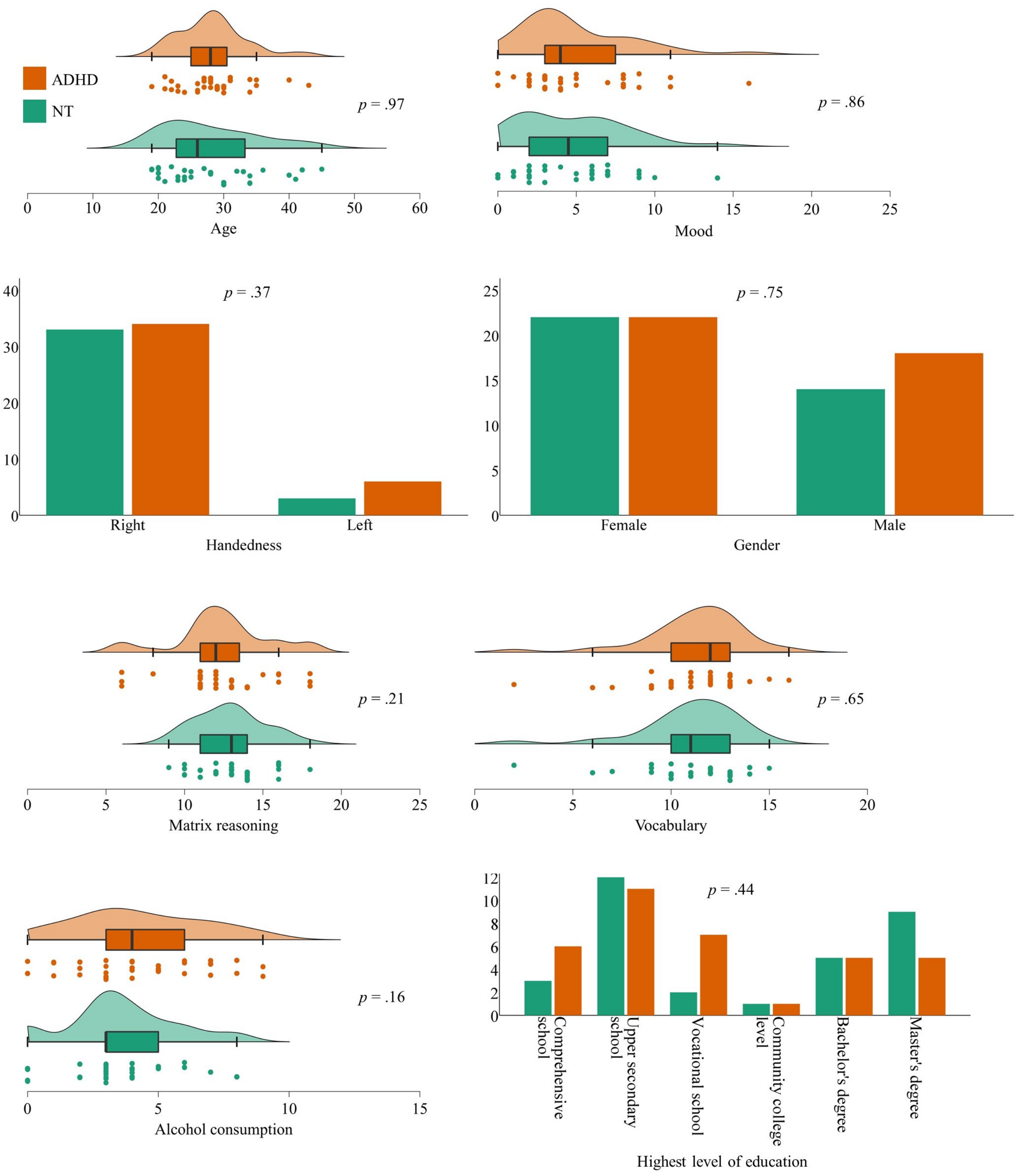

Supplementary figure 1. Demographic and clinical characteristics of the participants. Discrete variables are visualized with raincloud plots displaying individual data points, density plots and box plots. Categorical variables are illustrated with bar plots indicating number of participants in each category.

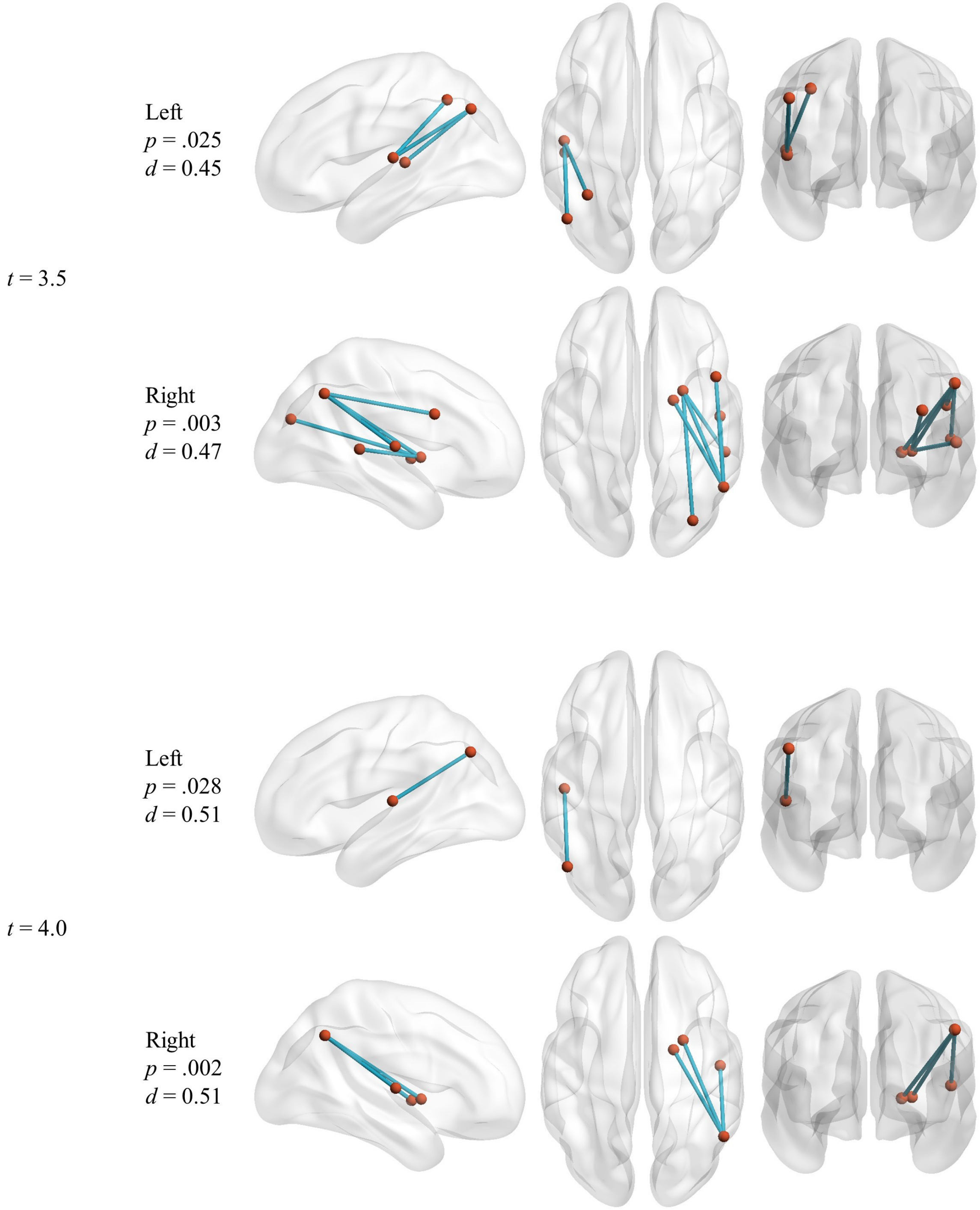

Supplementary figure 2. Networks identified with NBS distinguishing adults with and without ADHD with *t*-statistic thresholds 3.5 (upper rows) and 4.0 (bottom rows) using the Destrieux atlas. Edges represent decreased connectivity in the ADHD group compared to the NT adults. All *p*-values are FWE-corrected. With a threshold of 4.0, the left-sided network was identified only with intensity as a measure of network size. All other results are from using extend as a measure of network size.

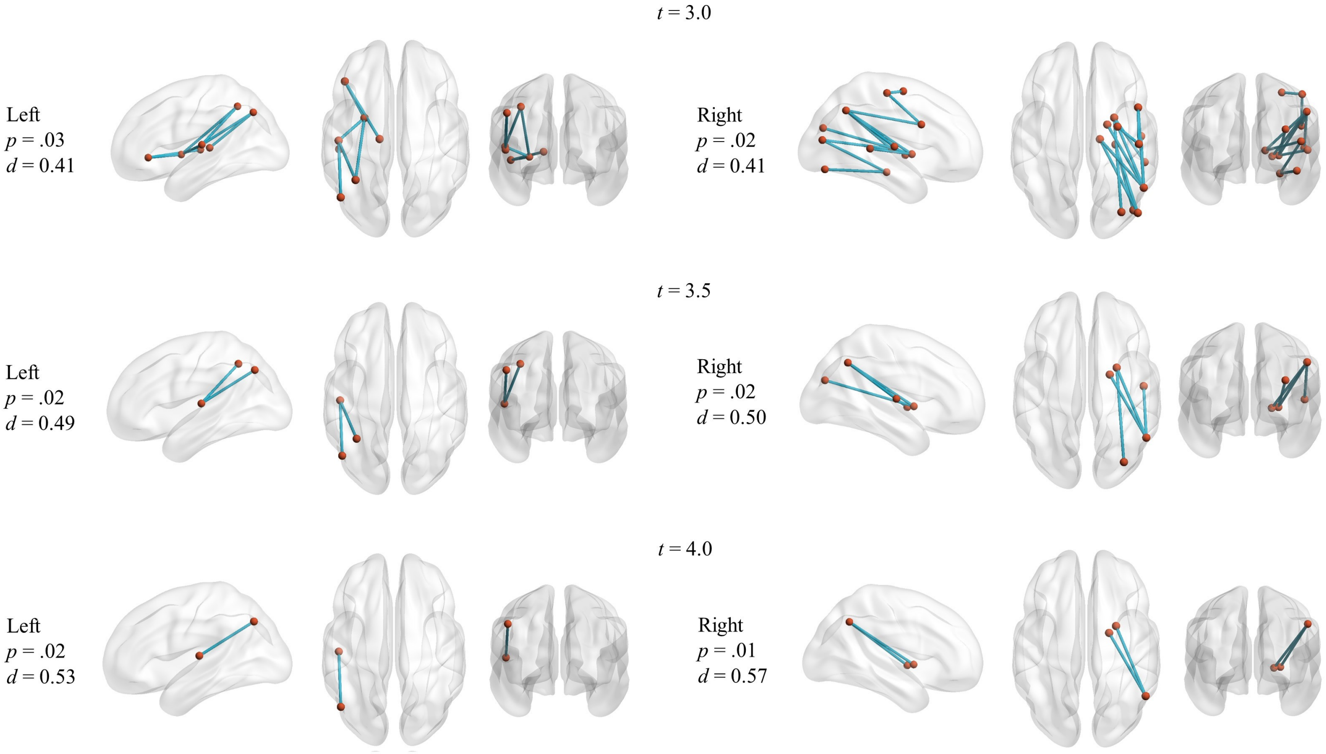

Supplementary figure 3. Results of the NBS group analysis after removing six outlier participants (edge weight > 2 SD in two or more connections in the NBS networks) from the NT group. Compared with the main results (see the main text), the subcallosal gyrus was no longer part of the left-sided network. In the right-sided network, two nodes (the intraparietal sulcus and middle occipital sulcus/lunate sulcus) were not present after outlier exclusion, and the inferior occipital sulcus appeared as a new node. The left-sided network was identified only with intensity as a measure of network size. Results of the right-sided network are presented by using extent as a measure of network size.

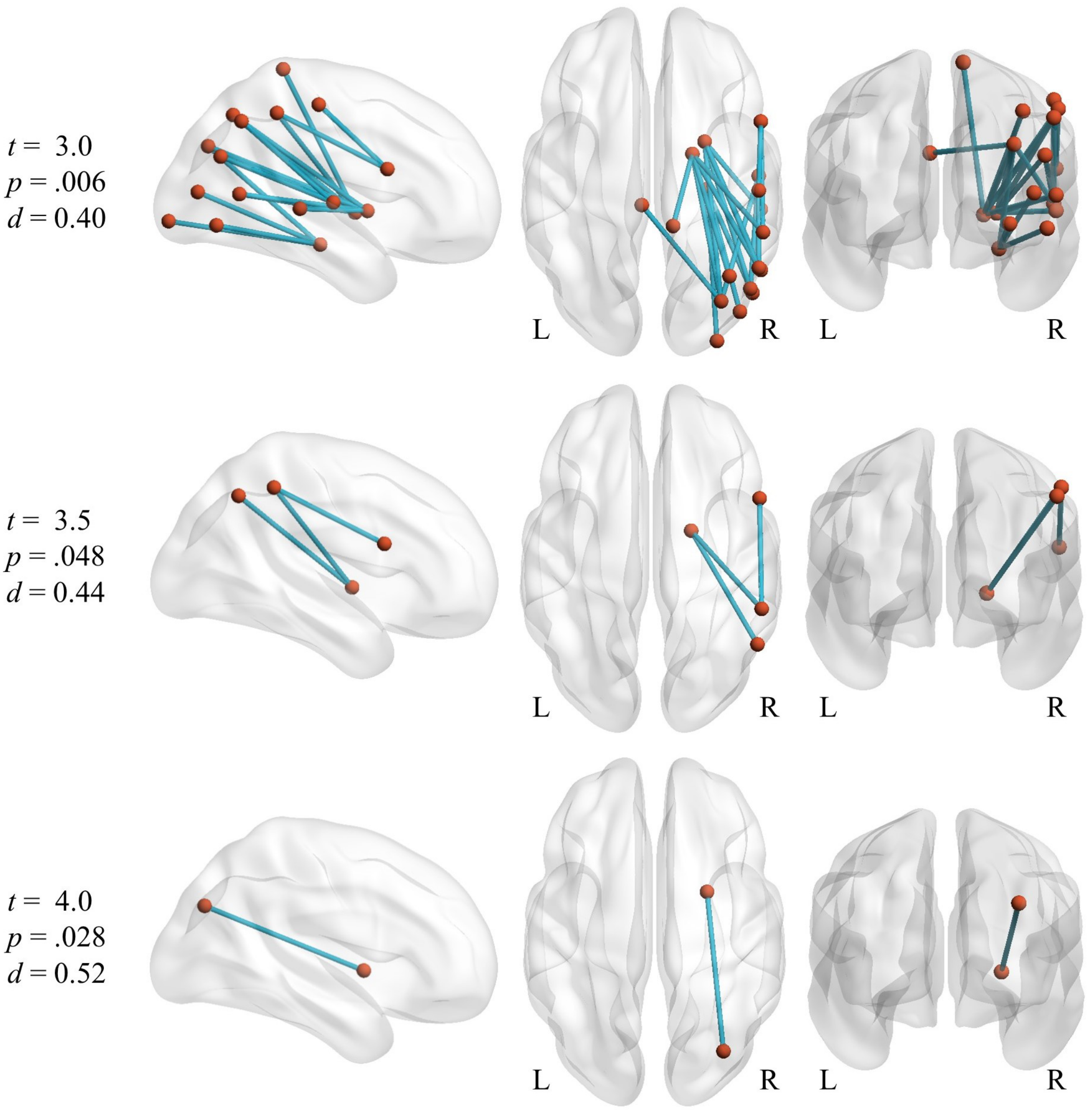

Supplementary figure 4. NBS results of the control analyses using the Schaefer parcellation. NBS identified decreased connectivity in the ADHD group as compared with the NT adults in a right-sided network largely overlapping with the network obtained with the Destrieux atlas. All *p*-values are FWE-corrected. With a threshold of 4.0, the network was identified only with intensity as a measure of network size. The other results are from using extend as a measure of network size. No statistically significant networks with ADHD > NT were identified (smallest *p*-value: $t = 4.0$, $p = .11$, FWE-corrected). No networks associated with the behavioural measures were found (smallest *p*-values: inattention: $t = 3.0$, $p = .09$; hyperactivity-impulsivity: $t = 3.5$, $p = .36$; commission errors: $t = 4.0$, $p = .09$; omission errors: $t = 3.0$, $p = .33$, FWE-corrected).

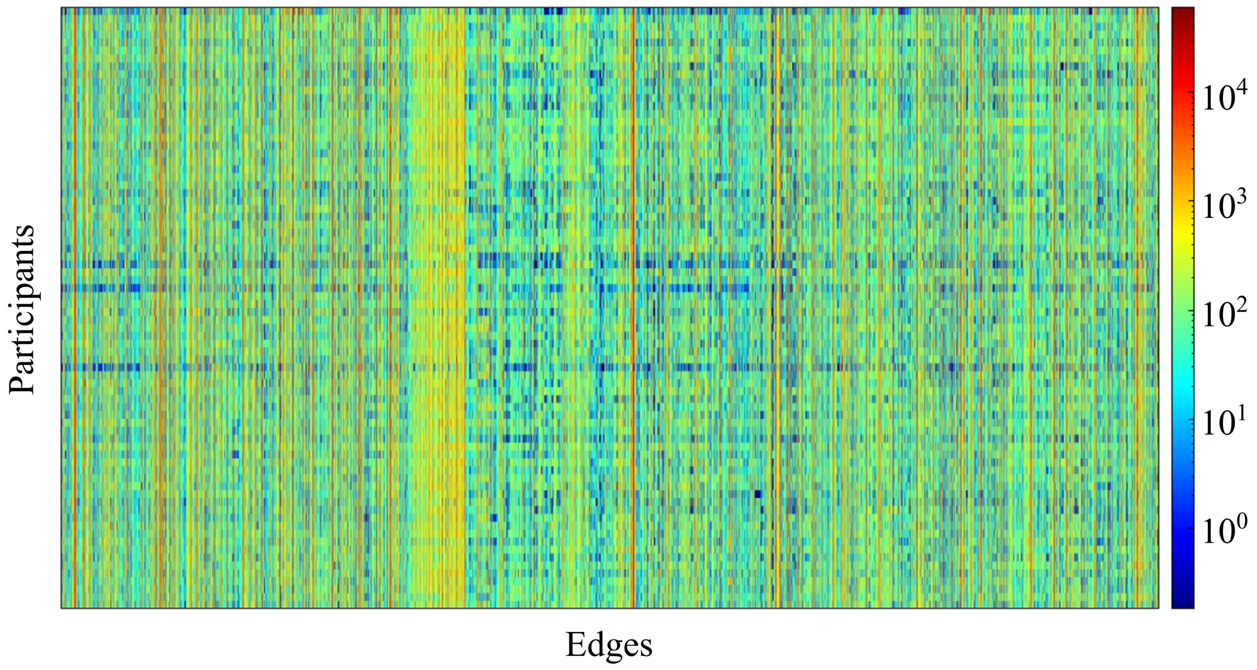

Supplementary figure 5. Heatmap figure of the edge weights of the whole connectome. Rows represent individual participants and columns represent connections between each two nodes in the parcellation. Colors illustrate the edge weights (i.e., number of streamlines) of the pairwise connections. Edges in which the edge weight was zero for all participants are omitted. Note that a logarithmic transformation was applied to the color scheme for visualization purposes.

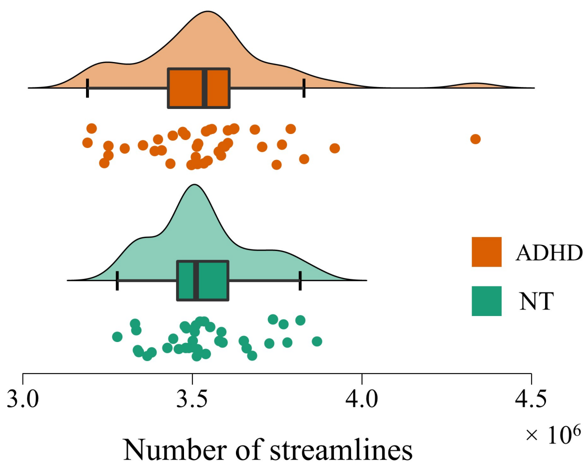

Supplementary figure 6. Total count of streamlines of the whole connectome by group. The raincloud plots display individual data points, density plots and box plots.

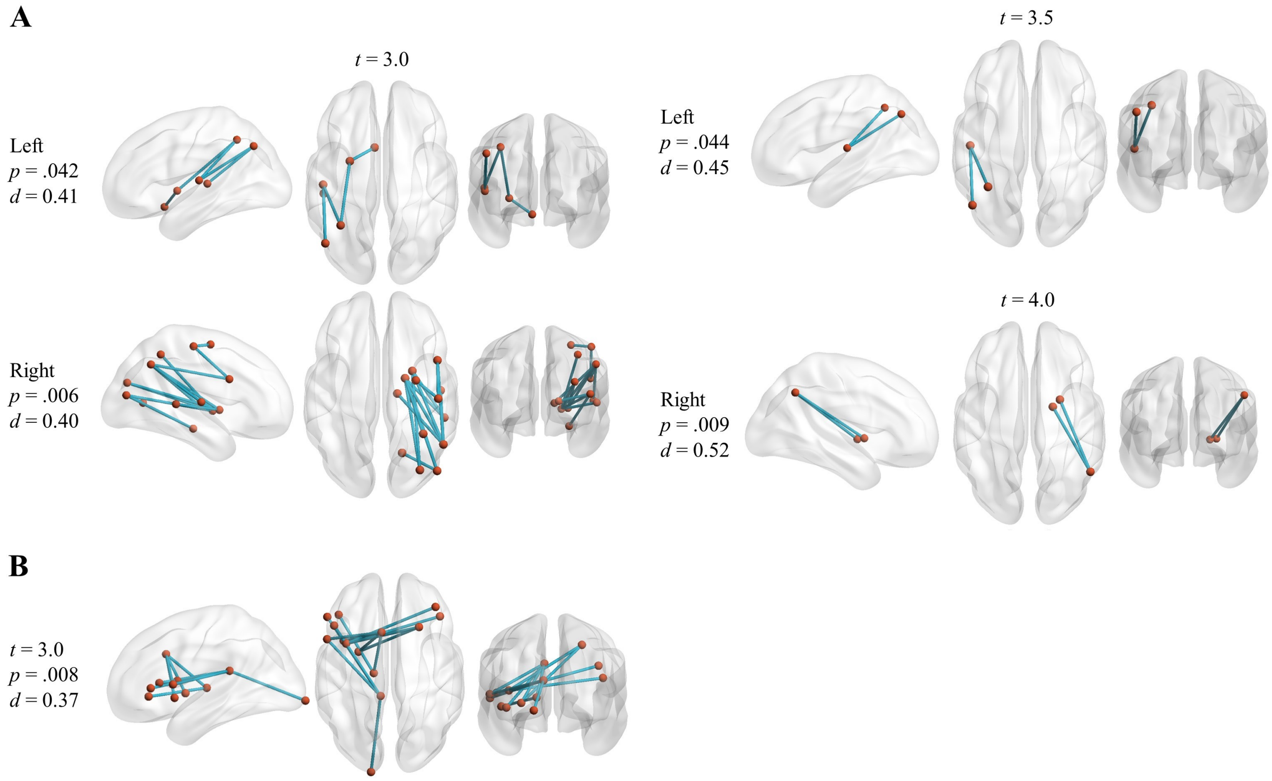

Supplementary figure 7. Results of the NBS analyses after removing one outlier participant (total count of streamlines > 3 SD) from the ADHD group. Only networks with differences compared with the main results are shown. (A) In the group analysis after the outlier exclusion, the anterior lateral sulcus and thalamus were no longer present in the left-sided network. In turn, in the right-sided network, the middle occipital sulcus/lunate sulcus node was not present after the outlier exclusion, and the calcarine sulcus appeared as a new node. The left-sided network was identified only with intensity as a measure of network size. Results of the right-sided network are presented by using extent as a measure of network size. (B) In the network representing hypoconnectivity associated with higher amount of commission errors in CPT-2, the left superior frontal gyrus and right putamen were not present after the outlier exclusion, and the right inferior frontal sulcus appeared as a new node.

Supplementary table 1. *Nodes and their center of mass MNI coordinates in the networks identified with NBS using the Destrieux atlas.*

Node	Center of mass coordinates (x, y, z)
Left-sided network, ADHD < NT	
Angular gyrus	-46, -67, 40
Subcallosal gyrus	-6, 11, -14
Anterior transverse temporal gyrus	-48, -19, 9
Anterior lateral sulcus (horizontal)	-43, 31, -3
Intraparietal sulcus	-34, -53, 45
Transverse temporal sulcus	-48, -27, 7
Thalamus	-13, -18, 5
Putamen	-26, 0, 0
Right-sided network, ADHD < NT	
Thalamus	13, -17, 5
Putamen	25, 1, 1
Pallidum	19, -4, 0
Hippocampus	26, -21, -15
Middle occipital gyrus	45, -79, 14
Angular gyrus	50, -58, 40
Postcentral gyrus	46, -21, 56
Anterior transverse temporal gyrus	48, -14, 8
Intraparietal sulcus	34, -49, 49
Middle occipital sulcus and lunate sulcus	34, -84, 5
Superior occipital sulcus and transverse occipital sulcus	31, -79, 24
Inferior precentral sulcus	45, 10, 28
Superior precentral sulcus	28, -6, 57
Superior temporal sulcus	51, -36, 6
Connectivity associated with commission errors in the CPT-2	
L aMCC	-7, 16, 33
L inferior frontal gyrus (opercular)	-53, 10, 8
L short insular gyri	-39, 9, -3
L superior frontal gyrus	-10, 31, 43
L superior circular sulcus	-37, 7, 11
L pericallosal sulcus	-8, -37, 19
L inferior frontal gyrus (triangular)	-52, 28, 5
L anterior lateral sulcus (vertical)	-47, 22, 9
L occipital pole	-16, -101, -6
L thalamus	-13, -18, 5
L anterior lateral sulcus (horizontal)	-43, 31, -3
L putamen	-26, 0, 0
R putamen	25, 1, 1
R middle frontal gyrus	38, 37, 31
R superior frontal sulcus	24, 20, 49

Note. L = left, R = right, aMCC = middle-anterior part of the cingulate gyrus and sulcus

Supplementary table 2. *Nodes of the NBS networks distinguishing adults with ADHD from NT controls identified with t-statistic thresholds 3.5 and 4.0 using the Destrieux atlas.*

Network		Node
$t = 3.5$		
	Left-sided	Angular gyrus
		Intraparietal sulcus
		Anterior lateral sulcus
		Anterior transverse temporal gyrus
	Right-sided	Putamen
		Pallidum
		Angular gyrus
		Anterior transverse temporal gyrus
		Superior occipital sulcus and transverse occipital sulcus
		Inferior precentral sulcus
		Superior temporal sulcus
$t = 4.0$		
	Left-sided	Angular gyrus
		Anterior transverse temporal gyrus
	Right-sided	Putamen
		Pallidum
		Angular gyrus
		Anterior transverse temporal gyrus

Supplementary table 3. *Group differences of global graph theory metrics obtained with the Destrieux atlas.*

Metric	ADHD<br>*M (SD)*	NT<br>*M (SD)*	*F(df)*	*p*
Betweennes centrality	540.28 (35.77)	544.35 (28.95)	0.29(1)	.59
Strength	43193 (2599)	43144 (1810)	0.01(1)	.93
Small-worldness	1.97 (0.42)	1.99 (0.54)	0.06(1)	.81
Normalized clustering coefficient	2.51 (0.12)	2.49 (0.09)	0.68(1)	.41
Normalized charasteristic path length	1.32 (0.20)	1.30 (0.19)	0.19(1)	.67
Normalized global efficiency	0.74 (0.03)	0.75 (0.02)	1.54(1)	.22
Normalized modularity	1.45 (0.02)	1.45 (0.02)	0.33(1)	.57

Note. M = mean, *SD* = standard deviation, *df* = degrees of freedom.

Supplementary table 4. *Correlations between ADHD symptoms, CPT performance and global graph theory metrics obtained with the Destrieux atlas.*

	Inattention		Hyperactivity-impulsivity		Commission errors		Omission errors	
Metric	ADHD group	All partici-pants	ADHD group	All partici-pants	ADHD group	All partici-pants	ADHD group	All partici-pants
Betweennes centrality	-.04	-.06	.28	.07	.06	-.11	.27	.11
Strength	.19	.14	.28	.15	-.15	-.01	.001	.09
Small-worldness	-.18	.10	.02	.07	.14	.09	.04	.02
Normalized clustering coefficient	-.25	-.01	.01	.06	.01	.13	.08	.10
Normalized charasteristic path length	.13	-.04	-.05	-.05	-.16	-.05	-.10	-.05
Normalized global efficiency	-.04	-.08	-.10	-.13	.09	-.05	.01	-.01
Normalized modularity	.11	.21	**.33***	.22	.09	.10	.03	.04

Note. * $p < .05$.

Supplementary table 5. *Nodes and their center of mass MNI coordinates in the networks identified with NBS using the Schaefer parcellation.*

Node	Center of mass coordinates (x, y, z)
R hippocampus	26, -21, -15
R putamen	25, 1, 1
R pallidum	19, -4, 0
R visual network area 5	48, -71, -6
R visual network area 8	31, -94, -4
R visual network area 11	42, -80, 10
R visual network area 15	33, -75, 32
R DAN posterior area 2	52, -60, 9
R DAN precentral ventral area 1	52, 11, 21
R somatomotor network area 1	51, -15, 5
R somatomotor network area 9	51, -22, 52
R somatomotor network area 17	10, -39, 69
R control network parietal area 2	53, -42, 48
R control network parietal area 3	37, -63, 47
R DMN parietal area 1	47, -69, 27
R DMN parietal area 3	51, -59, 44
R DMN temporal area 5	52, -31, 2
L control network cinculate area 1	-5, -29, 28

Note. R = right, L = left, DAN = dorsal attention network, DMN = default mode network

Supplementary table 6. *Group differences of global graph theory metrics obtained with the Schaefer parcellation.*

Metric	ADHD *M (SD)*	NT *M (SD)*	*F(df)*	*p*
Betweennes centrality	743.25 (42.04)	750.61 (42.16)	0.58(1)	.45
Strength	31588 (2257)	31526 (1444)	0.02(1)	.89
Small-worldness	2.00 (1.20)	1.84 (0.73)	0.52(1)	.48
Normalized clustering coefficient	3.20 (0.15)	3.18 (0.13)	0.76(1)	.39
Normalized charasteristic path length	1.80 (0.40)	1.85 (0.32)	0.26(1)	.62
Normalized global efficiency	0.61 (0.02)	0.61 (0.01)	0.01(1)	.93
Normalized modularity	1.57 (0.03)	1.58 (0.03)	0.33(1)	.57

Note. M = mean, *SD* = standard deviation, *df* = degrees of freedom.

Supplementary table 7. *Correlations between ADHD symptoms, CPT performance and global graph theory metrics within the ADHD group obtained with the Schaefer parcellation.*

Metric	Inattention	Hyper-activity-impulsivity	Commission errors	Omission errors
Betweennes centrality	.04	-.05	-.04	-.07
Strength	.18	.27	-.13	-.001
Small-worldness	-.22	-.18	.08	-.08
Normalized clustering coefficient	-.30	.02	.03	.13
Normalized charasteristic path length	.23	.08	-.10	-.02
Normalized global efficiency	-.05	-.18	.05	-.09
Normalized modularity	-.11	.03	-.11	.11

Note. * $p < .05$.

Supplementary table 8. *Participants' mean range of movement during diffusion-weighted imaging. Standard deviations, and minimum and maximum values in parentheses.*

Variable	ADHD group ($N = 40$)	Neurotypical group ($N = 36$)
RMS movement, mm		
relative to the first volume	0.78 (0.46, 0.38–2.86)	0.78 (0.59, 0.37–3.71)
relative to the previous volume	0.22 (0.16, 0.10–1.00)	0.21 (0.07, 0.11–0.47)
Restricted RMS movement, mm		
relative to the first volume	0.92 (0.79, 0.24–4.85)	1.00 (0.93, 0.27–5.27)
relative to the previous volume	0.18 (0.23, 0.07–1.31)	0.14 (0.10, 0.07–0.48)

Note. RMS = root mean square.
